# A Novel Dopamine Electrochemical Sensor Based on Pt/CNTs-N-S/Electrode

**DOI:** 10.3390/s26061879

**Published:** 2026-03-17

**Authors:** Pingping Yang, Zhaopu Li, Jinpu Xie, Yukun Tang, Yinchen Liu, Lingxin Zhou, Tengfei Duan, Zhonghui Deng, Siwen Du, Qifei Zhang, Yabing Lu, Jingjing Du, Lijian Xu

**Affiliations:** 1 School of Biological Science and Medical Engineering, Hunan University of Technology, Zhuzhou 412007, China; 2 School of Packaging Engineering, Hunan University of Technology, Zhuzhou 412007, China; 3 College of Science and Technology, Hunan University of Technology, Zhuzhou 412007, China; 4 School of Materials Science and Engineering, Hunan University of Technology, Zhuzhou 412007, China

**Keywords:** S, N-doped, multiwalled carbon nanotube, Pt nanoparticles, sensor, dopamine

## Abstract

Dopamine (DA) plays an extremely crucial role in the metabolic processes of the human body. Accurate detection of DA is of great significance for many major diseases. This study reports an innovative synthesis method for composite material in which sulfur (S) and nitrogen (N) are incorporated into multi-walled carbon nanotubes (MWCNTs), and platinum (Pt) nanoparticle sensors (Pt/CNTs-N-S) are loaded for the highly sensitive and selective electrochemical detection of DA. The linear range of this sensor is from 0.0078 to 2 mM, and the limit of detection (LOD) is 0.73 μM (S/N = 3) for DA detection. The outstanding detection performance exhibited by Pt/CNTs-N-S is mainly attributed to the co-doping of N and S, which improves the surface properties of MWCNTs, and the dispersion of Pt nanoparticles (5.22 nm), which significantly increases the electrochemically active surface area (ESCA). In addition, the Pt/CNTs-N-S sensor also exhibits excellent stability and anti-interference performance. Overall, this study provides a simple and practical strategy for the potential application of Pt-based sensors in the detection of DA.

## 1. Introduction

Dopamine (DA), a crucial neurotransmitter, plays a pivotal role in the central nervous system, regulating various physiological functions including mood, cognition, and motor control [1,2,3]. Abnormal levels of DA are closely associated with numerous neurological disorders such as Parkinson’s disease, Schizophrenia, and Depression [4,5]. Therefore, the development of reliable, sensitive, and selective methods for DA quantification is of paramount significance in clinical diagnosis and neurochemical research [6,7,8]. Among various analytical techniques (chromatography, mass spectrometry, spectroscopy, electrochemical method and so on), electrochemical sensors have garnered tremendous attention due to their inherent advantages of high sensitivity, rapid response, simple operation, and low cost [9,10,11].

In electrochemical sensors, the material of the modified electrode plays a crucial role in the response to DA [12]. As an electrochemical method, the selection and optimization of sensing materials are also key factors influencing the sensing performance [13,14]. Multi-walled carbon nanotubes (MWCNTs), as a kind of nanomaterial with unique structure and properties, are widely applied due to their excellent electrical conductivity, high specific surface area, good thermal stability and chemical stability [15,16,17]. These characteristics give them a significant advantage in enhancing the performance of electrochemical sensors. However, disadvantages such as poor dispersion and inaccurate sensing sensitivity have restricted its wide application [18]. So far, scholars have adopted strategies such as metal loading, chemical modification, element doping, and material composites to enhance their electrochemical performance [19,20,21]. Recently, the *co*-doping of two heteroatoms, such as S and N, has been demonstrated to induce a synergistic effect, generating more pronounced electronic modulation and structural defects than single doping, thereby further boosting the electrocatalytic activity [22,23,24]. For instance, Wu et al. successfully fabricated a one-dimensional N-doped porous carbon nanotube array (1D CNTs@NPC) that exhibited excellent electrochemical response to DA [23]. Korusenko et al. prepared a titanium-based S-doped carbon nanotube (S-MWCNTs/Ti) electrocatalyst, demonstrating that S doping enhanced the electrochemical performance of the catalyst [24]. At present, there are relatively few studies on synthesizing S and N dual-doped carbon nanomaterials based on conductive polymers. Especially in terms of using this material for electrochemical detection of DA, the research is even more scarce [25].

In order to further enhance the electrochemical analysis performance of MWCNTs, various electrode modification materials have been designed and utilized to strengthen the electrochemical signals, such as noble metal nanoparticles (Au, Pt, and Pd) and molybdenum disulfide (MoS_2_), zinc oxide (ZnO), silicon dioxide (SiO_2_), etc. [26,27,28,29]. For instance, Feng et al. synthesized a COF/Pt/MWCNT-COOH nanocomposite material for the sensitive detection of DA using electrochemical sensors [30]. Zhang et al. successfully constructed a Pt@g-C_3_N_4_/N-CNTs nanocomposite material, which exhibited a sensitive electrochemical response behavior to DA [31]. The Pt nanoparticles can be uniformly dispersed on the surface of MWCNT, increasing the specific surface area and the number of active sites of the electrode [32]. More active sites mean that more DA molecules can be adsorbed, thereby enhancing the sensor’s ability. In conclusion, loading Pt in the form of nanoparticles onto the S- and N-doped CNTs of the conductive polymer increases the specific surface area and also enhances its adsorption capacity, thereby improving the sensitivity of the sensor [33,34,35].

This study reports a novel synthesis method for a new type of composite material, which involves incorporating sulfur and nitrogen into multi-walled carbon nanotubes (Pt/CNTs-N-S) and then loading Pt nanoparticles to obtain the material. It is used for high-sensitivity and selectivity in DA electrochemical detection. The CNTs-N-S matrix serves not only as a highly conductive scaffold, but also, more importantly, it provides abundant anchoring sites for the uniform dispersion and stabilization of Pt nanoparticles. The electronic synergy between S, N *co*-dopants and Pt nanoparticles is expected to significantly enhance the electrocatalytic activity towards the oxidation of DA. The as-prepared Pt/CNTs-N-S-modified electrode demonstrates outstanding analytical performance, including a wide linear range and a remarkably low detection limit. The performance of Pt/CNTs-N-S in detecting DA was evaluated through several electrochemical tests.

## 2. Experimental Methods

### 2.1. Experimental Materials

MWCNTs (outer diameter: 8 nm; length: 10–30 μm), chloroplatinic acid hexahydrate (H_2_PtCl_6_·6H_2_O, 99%), sodium dodecyl sulfate (SDS, 98%), Ploy 3,4-ethylenedioxythiophene (PEDOT, 98%), Polyaniline (PANI, 98%), ammonium persulfate, and dopamine hydrochloride were purchased from Beijing Inokai Technology Co., Ltd. (Beijing, China). Sulfuric acid (H_2_SO_4_, 98%) and nitric acid (HNO_3_, 68%) were purchased from Hunan Huihong Reagent Co., Ltd. (Changsha, China), and Sodium borohydride (NaBH_4_, 98%) was obtained from China National Pharmaceutical Chemical Reagent Co., Ltd., Shanghai, China. The phosphate-buffered solution (PBS, 0.2 M) is composed of disodium hydrogen phosphate (NaH_2_PO_4_, 0.2 M) and dihydrogen phosphate disodium (Na_2_HPO_4_, 0.2 M).

### 2.2. Methods

#### 2.2.1. Preparation of Acidified MWCNTs

First, 0.5 g of MWCNTs was added to a mixture of 30 mL of concentrated H_2_SO_4_ and HNO_3_ (with a volume ratio of 1:1). Then, this was refluxed at 140 °C for 3 h. The sample was repeatedly washed with water until the pH was neutral and then dried at 80 °C. The acidified MWCNTs were obtained and labeled as CNTs-AO.

#### 2.2.2. Preparation of Pt/CNTs

Next, 10 mg of MWCNTs-AO was added to 20 mL of deionized water before undergoing ultrasonic stirring and dispersing. Then, 0.024 mL of the hexahydrate chloroplatinic acid (H_2_PtCl_6_·6H_2_O) saline, prepared with deionized water (containing 2.5 mg of Pt), was added. Finally, 100 mg of sodium borohydride was added. The mixture was reacted at 80 °C for 6 h. After cooling, it was washed alternately with water and ethanol, filtered, and dried under vacuum at 60 °C to obtain the catalyst-labeled Pt/CNTs.

#### 2.2.3. Preparation of Pt/CNTs-N-S

Next, 2.307 g of SDS was dissolved in 20 mL of water and stirred to dissolve. Then, 180 μL of PEDOT and 120 μL of PANI were added to the above saline and vigorously stirred for 2 h. The saline was quickly injected into (NH_4_)_2_S_2_O_8_ saline (20 mL, 0.04 M) and then stirred for 24 h. The sample was washed at 60 °C and dried overnight to obtain the PEDOT-PANI-coated MWCNT composite material. Subsequently, the obtained PEDOT-PANI/CNTs were calcined at 800 °C for 3 h. The final product was named Pt/CNTs-N-S.

### 2.3. Materials Characterization

The morphology of the products was observed via transmission electron microscopy (TEM). Their crystal structures and elemental composition were analyzed using X-ray diffraction (XRD, Bruker D8, Karlsruhe, Germany). Conventional phase scanning was performed within the range of 10° to 80°, with a continuous scan rate of 2°/min and X-ray photoelectron spectroscopy (XPS, Thermo Kalpha, Waltham, MA, USA).

### 2.4. Electrochemical Testing of DA

To evaluate the performance of Pt/CNTs-N-S, a three-electrode system, which consisted of a platinum working electrode, a bare glassy carbon reference electrode (GCE), and a saturated calomel reference electrode (SCE), was used. The preparation steps of the Pt/CNTs-N-S/GCE are as follows: Firstly, the GCE is pre-treated. Then, it is polished successively with 5.0, 1.0 and 0.3 μm of Al_2_O_3_ powder. Subsequently, it is ultrasonically cleaned with water and ethanol for 10 min. Finally, 5 μL of the Pt/CNTs-N-S saline (600 μL of ethanol, 3 mg of Pt/CNTs-N-S catalyst, and 5 μL of Nafion) is dropped onto the pre-treated GCE and dried in a desiccator to obtain the Pt/CNTs-N-S/GCE. The CHI660E electrochemical workstation (Shanghai Chenhua, Shanghai, China) was employed. Cyclic voltammetry (CV, initial potential: 0.6 mV, high potential: −0.6 mV, scan rate: 50 mV/s, quiet period: 2 s), Electrochemical Impedance Spectroscopy (EIS, 0.1 Hz–100 kHz, 10 mV), differential pulse voltammetry (DPV, the pulse amplitude is 50 mV, the pulse width is 50 ms, and the step potential is 2 mV), and other techniques were carried out in a 10 mL electrochemical cell containing 0.2 M phosphate-buffered saline (PBS, PH = 7.0) with an appropriate concentration of DA.

## 3. Results and Discussion

### 3.1. Characterization and Analysis of Pt/CNTs-N-S Materials

**Scheme 1:**
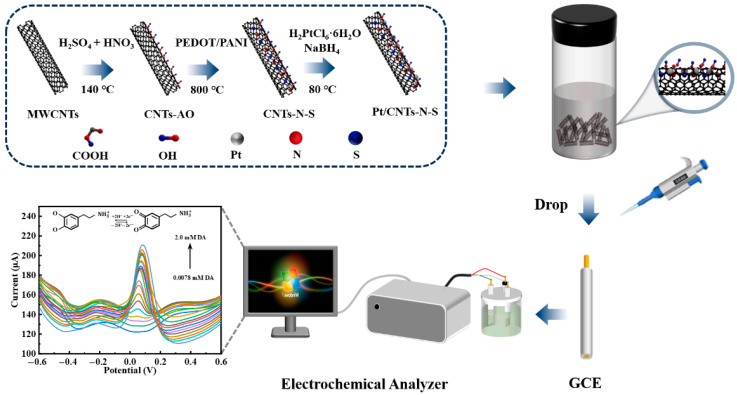
Schematic diagram of Pt/CNTs-N-S synthesis and electrochemical detection of DA.

In the electrode preparation, as shown in Scheme 1, MWCNTs-AO (CNTs) were doped with S and N (CNTs-N-S). In the aqueous suspension of CNTs-N-S, after injecting H_2_PtCl_6_·6H_2_O, Pt was reduced by NaBH_4_ and was then loaded on the surface of CNTs-N-S, resulting in Pt/CNTs-N-S. The synergistic effect of Pt NPs and S- and N-doped CNTs (CNTs-N-S) significantly enhanced the electronic conductivity of the electrode, thereby achieving highly sensitive detection of DA.

**Figure 1:**
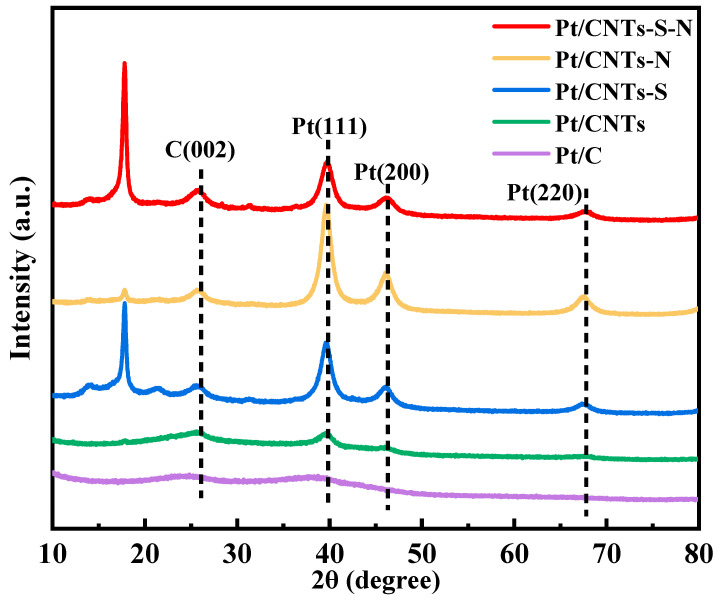
XRD patterns of all samples.

Figure 1 displays the XRD patterns of all materials, Pt/CNTs-N-S, Pt/CNTs-S, Pt/CNTs-N, Pt/CNTs and Pt-C. The C (002) crystal plane diffraction peak at 2θ = 26.2° corresponds to the layered structure of MWCNTs. This characteristic C (002) peak is present in all samples, indicating the integrity of the carrier structure. The three typical Pt diffraction peaks observed, Pt (111), (200), and (220), appear near 39.8°, 46.4°, and 67.6°, respectively, consistent with the FCC (face-centered cubic) structure standard card for metallic Pt (PDF#04-0802) [36]. Among the five materials, all the Pt peaks of Pt/CNTs-N-S exhibited higher intensity and sharper peak shapes, indicating that the main reason was the strong synergistic effect of S and N *co*-doping. This substantially enhanced the crystallinity and grain size of Pt particles, potentially forming more ordered Pt nanostructures. Next in intensity were the Pt peaks of Pt/C, followed by Pt/CNTs-N. The Pt peaks in the two single-doped samples (Pt/CNTs-S and Pt/CNTs-N) were markedly higher than those in the undoped Pt/CNTs but lower than those in the double-doped Pt/CNTs-N-S. This indicates that both S and N contribute to improving Pt distribution, though their effects are weaker than the peak intensity enhancement achieved through simultaneous doping.

**Figure 2:**
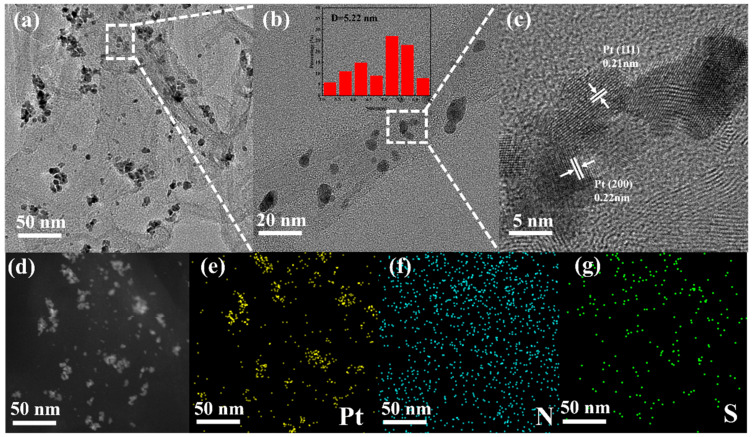
(**a**,**b**) TEM of Pt/CNTs-N-S (inset of (**b**)); particle size chart of Pt; (**c**) HRTEM of Pt/CNTs-N-S; (**d**–**g**) element mapping diagram of Pt, N, and S in the Pt/CNTs-N-S.

To observe the size and morphology of the Pt/CNTs-N-S, as well as the loading state of Pt, we conducted TEM testing, which clearly reveals the presence of CNTs exhibiting typical tubular structures with diameters typically ranging from 10 to 30 nm (Figure 2a). This indicates that the CNTs maintained excellent morphological and structural integrity during preparation, providing the foundation for the composite material’s superior mechanical and electrical properties. TEM and HR-TEM images of Pt/CNTs-N-S (Figure 2b) clearly reveal Pt nanoclusters loaded onto the CNTs. The Pt (111) interplanar spacing in Pt/CNTs-N-S is 0.21 nm (Figure 2c), corresponding to the Pt (111) crystal plane, indicating good crystallinity of the Pt nanoparticles. As shown in the inset of Figure 2b, the average particle size of the loaded Pt nanoparticles is 5.22 nm, indicating that the Pt nanoparticles are uniformly loaded onto CNTs-N-S. The elemental distribution map in Figure 2d–g clearly and intuitively shows that S and N are uniformly distributed within the CNTs, indicating successful doping of S and N into the CNTs. The EDX analysis of Pt/CNTs-N-S materials is shown in Figure S1. The TEM and mapping diagrams of Pt/CNTs-N and Pt/CNTs-S materials are shown in Figures S2 and S3. All the Pt nanoparticles are evenly distributed on the surface of CNTs, indicating that Pt has been successfully loaded.

**Figure 3:**
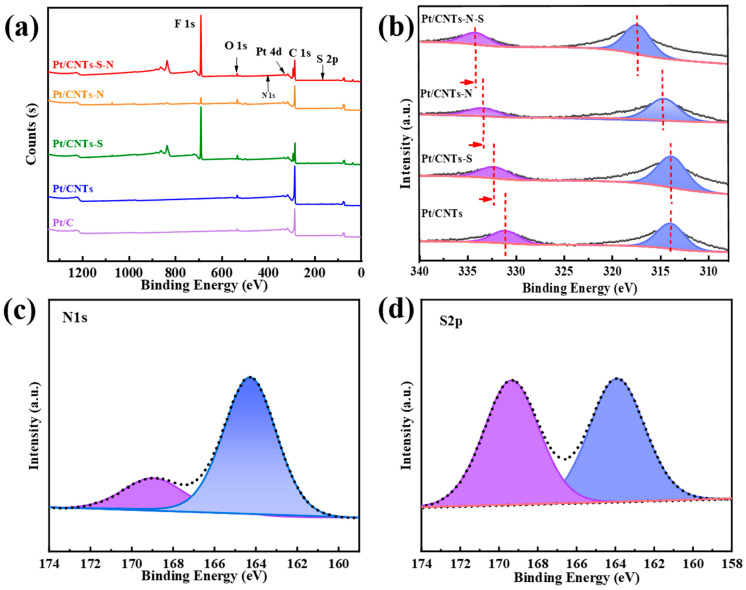
(**a**,**b**) The full-spectrum XPS and Pt4d spectra of Pt/CNTs-N-S, Pt/CNTs-N, Pt/CNTs-S, Pt/CNTs-N; (**c**) the N 1s spectrum of Pt/CNTs-N-S; (**d**) the S 2p spectra of Pt/CNTs-N-S.

The elemental composition and chemical structure of the catalytic surface elements were characterized by XPS. Figure 3a shows the XPS spectra of Pt/CNTs-N-S, Pt/CNTs-S, Pt/CNTs-N, Pt/CNTs, and Pt-C. XPS signal peaks for C 1s (284.8 eV), O 1s (531.2 eV), and Pt 4d (315.0 eV) are observed. Additionally, S 2p (164.3 eV, 168.9 eV) for Pt/CNTs-N-S and Pt/CNTs-S further confirms the covalent doping of sulfur (S^2^) and nitrogen (e.g., pyridine/amide nitrogen) on the carbon tube surface, which enhances electron transport and surface activity. The Pt (4d) spectrum for Pt/CNTs, Pt/CNTs-N, Pt/CNTs-S, and Pt/CNTs-N-S (Figure 3b) presents different peaks that indicate the existence of different Pt oxidation states. For Pt/CNTs-N-S, the binding energies of metallic Pt (0) and Pt (+2) states were 316.9 eV (Pt4d_5/2_) and 334.6 eV (Pt4d_3/2_). Similarly, for Pt/CNTs-N, the binding energies of 315.3 eV (Pt4d_5/2_) originate from the metal Pt (0), while the binding energies of 334.5 eV (Pt4d_3/2_) come from the Pt (+2) state. For Pt/CNTs-S, the binding energies of 314.8 eV (Pt4d_5/2_) and 332.4 eV (Pt4d_3/2_) correspond to the metallic Pt (0) and Pt (+2), respectively. For Pt/CNTs, the 314.7 eV (Pt4d_5/2_) and 331.4 eV (Pt4d_3/2_) binding energies are associated with the metallic Pt (+2) and Pt (0) states. The comparative analysis indicates that the peak of the 4d energy level of Pt has shifted, suggesting that the doping of sulfur and nitrogen has affected the formation of Pt-O bonds and changed the electronic structure [37,38]. This is also supported by the calculated results of XPS (Table S1), where the area ratio of Pt (0) over Pt (+2) in Pt/CNTs-N-S (0.63) is greater than that of Pt/CNTs-S (0.61), Pt/CNTs-N (0.60) and Pt/CNTs (0.59). Figure 3c,d demonstrate that the N1s (400.48 eV) and S2p (164.18 ev,169.32 eV) spectra confirm that sulfur (S^2−^) and nitrogen (such as pyridine/amide nitrogen) are covalently doped onto the carbon tube surface, enhancing electron transport and surface activity [39].

### 3.2. Electrode Modification and Electrochemical Studies

#### 3.2.1. Different pH Optimization

**Figure 4:**
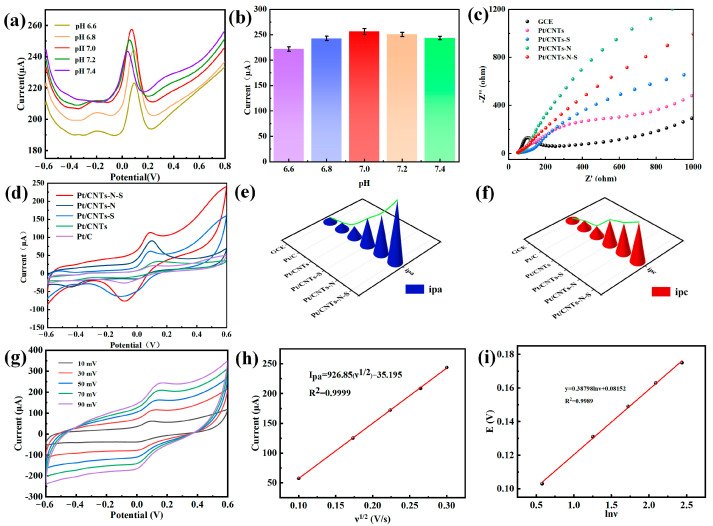
(**a**) DPV of Pt/CNTs-N-S at different pH values in 0.2 M PBS containing DA; (**b**) DA response current at different pH values; (**c**) EIS measurements of several electrodes in a 5 mM K_3_[Fe(CN)_6_] saline; (**d**) CV of GCE, Pt-C, Pt/CNTs and Pt/CNTs-N-S in the presence of DA; (**e**,**f**) data graph of oxidation–reduction peaks of different materials; (**g**) CV curves of the response of Pt/CNTs-N-S to DA at different scanning rates electrodes; (**h**) the linear relationship between peak current and the root of scanning rate; (**i**) the linear relationship between potential and scan logarithm.

Firstly, the detection performance of the modified electrode Pt/CNTs-N-S was evaluated in PBS solutions with pH values of 6.6, 6.8, 7.0, 7.2 and 7.4. Figure 4a shows that the differential pulse voltammogram (DPV; the pulse amplitude is 50 mV, the pulse width is 50 ms, and the step potential is 2 mV) response value of DA on the Pt/CNTs-N-S electrode in the PBS solution with pH = 7.0 is the highest. The results show that when the PBS solution (pH = 7.0) is used, the uniform distribution of Pt nanoparticles on the surface of CNTs increases the active area of the electrode and also enhances its adsorption capacity for DA, allowing more DA molecules to approach the electrode surface and participate in the reaction. Subsequent tests were conducted under this condition. The corresponding currents of the working electrode to DA at different pH values are shown in Figure 4b. After repeated comparisons, we found that when the pH value of PBS was 7.0, the Pt/CNTs-N-S sensor had the highest response value.

#### 3.2.2. Electrochemical Property Analysis

The error bar data were obtained through three sets of parallel experiments. As shown in Figure 4c, the electrodes were studied for their conductivity and other properties in a 5 mM K_3_[Fe(CN)_6_] solution using electrochemical impedance spectroscopy (EIS, 0.1 Hz–100 kHz, 10 mV). The bare GCE is shown as a semi-circle, indicating that the bare GCE has the characteristics of high resistivity and low conductivity. Compared with Pt/CNTs (63 Ω), Pt/CNTs-S (58 Ω), and Pt/CNTs-N (53 Ω), Pt/CNTs-N-S (50 Ω) has a higher conductivity. The S or N doping of carbon nanotubes essentially alters the electronic structure and surface chemical properties of the carbon framework, thereby enabling Pt/CNTs-N-S (50 Ω) to exhibit higher conductivity and lower charge resistance. This result indicates that the resistivity of Pt/CNTs-N-S is lower, allowing electrons to transfer more easily to the surface of the modified electrode and participate in the reaction with DA. Compared with Pt/CNTs, the conductivities of doped materials Pt/CNTs-S, Pt/CNTs-N and Pt/CNTs-N-S are higher, suggesting that the doping of S and N can significantly improve the detection performance.

#### 3.2.3. The Electrochemical Properties of the DA Electrode

We measured the DA response signals of each material using the CV method at a sweep speed of 40 mV. As shown in Figure 4d, in the PBS (0.2 M) containing 1 M DA, all the electrodes, namely Pt/C, Pt/CNTs, Pt/CNTs-N, Pt/CNTs-S and Pt/CNTs-N-S, exhibited typical characteristic oxidation peaks of DA, which were located at approximately 0.1 V. Thus, it can be concluded that these electrodes have catalytic effects on the oxidation reaction of DA and can effectively detect the presence of DA. Under the same conditions, compared with the above-mentioned Pt/C and other types of electrodes, the current response of dopamine to the Pt/CNTs-N-S electrode is significantly enhanced. The cyclic voltammogram results in Figure 4e,f show that the Pt/CNTs without S and N doping only present low-amplitude redox peaks, indicating that the surface charge transfer kinetics is limited and the density of catalytic sites is insufficient; meanwhile, the Pt/CNTs-N-S electrode after S-N *co*-doping shows significantly enhanced anode/cathode peak currents within the same potential window, and the peak shape is sharper and more symmetrical. This indicates that the doping of Pt, S, and N synergistically enhanced the electrocatalytic performance of CNTs for DA [40,41]. The electrochemically active surface areas of several modified electrodes were compared, and the specific method is shown in Figure S4. The final calculated ECSA values for Pt/CNTs-N-S, Pt/CNTs-N, Pt/CNTs-S, Pt/CNTs and Pt/C were 35.2 m^2^ g^−1^, 27.1 m^2^ g^−1^, 8.5 m^2^ g^−1^, 7.2 m^2^ g^−1^ and 5.1 m^2^ g^−1^, respectively. Among them, the Pt/CNTs-N-S/GCE has the highest electrochemically active area.

It can be seen from Figure 4g that at a potential of 0.23 V, as the scanning rate increases from 10 to 90 mV/s, the peak current gradually increases. There is a good linear relationship between the peak current obtained through linear fitting and the scanning rate (Figure 4h), which is represented by Ipa = 926.86v^1/2^ − 35.195 (R^2^ = 0.9999), indicating that the electrocatalytic oxidation of DA on the Pt/CNTs-N-S/GCE surface is a diffusion-controlled process. The (Epa, Epc) and (Ipa, Ipc) of Pt/CNTs-N-S at different sweep rates are shown in Figures S5 and S6. Furthermore, the oxidation peak potential shows a linear relationship with the logarithm of the sweep rate, and the linear relationship is given by y = 0.38978 ln(v) + 0.08152 (R^2^ = 0.9989) (Figure 4i). This indicates that the DA reaction system is controlled by diffusion when the Pt/CNTs-N-S/GCE working electrode is used. According to the formula:

*P* = *P*° + (*RT*/αn*F*)ln(*RTk*°/αn*F*) + (*RT*/αn*F*)ln*v*

where *T* is for the Kelvin temperature, *R* is for the ideal gas constant, *k*° is for the heterogeneous electron transfer rate, *F* is for the Faraday constant, *P*° is for the formal potential, a is 0.5 for a quasi-reversible process, and *n* is for the number of electrons. Then, n is calculated to be approximately 2. Therefore, the oxidation of dopamine is a quasi-reversible process of electron transfer.

#### 3.2.4. The Detection of Different Concentrations of DA and the Anti-Interference Ability of Pt/CNTs-N-S/GCE

**Figure 5:**
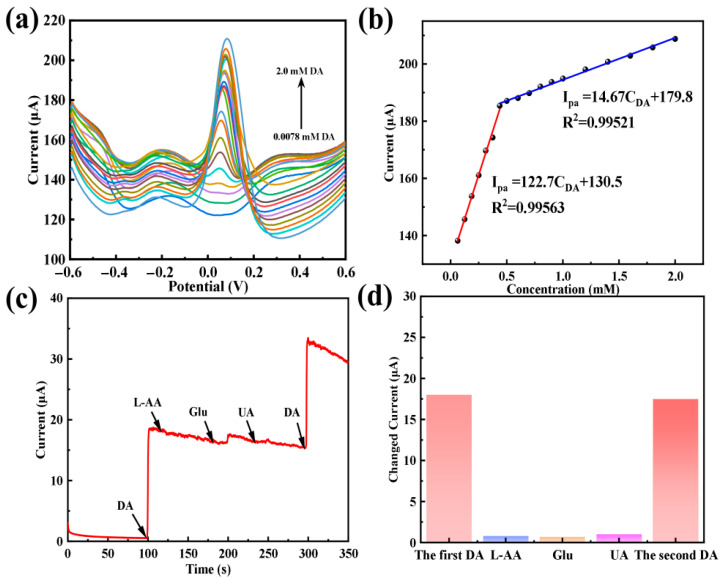
(**a**) DPV curves of Pt/CNTs-N-S/GCE response to different concentrations of DA saline; (**b**) linear relationship between the peak current value and the concentration of DA; (**c**) I-t curve in the presence of various interfering substances; (**d**) the corresponding changed current value as the addition of DA and interfering substances.

The DPV responses of Pt/CNTs-N-S under different concentrations of DA were quantitatively analyzed. As shown in Figure 5a, the *Pox* significantly increased with the increase in DA concentration, indicating that the sensor has excellent electrochemical response sensitivity to DA. Figure 5b presents a two-segment linear relationship between *Pox* and DA concentration, and the corresponding linear regression equations are I_pa_ = 122.7C_DA_ + 130.5 (R^2^ = 0.99563) and I_pa_ = 14.67C_DA_ + 179.8 (R^2^ = 0.99521), indicating good linear response characteristics in both high and low concentration ranges. The corresponding LOD was calculated as 0.73 μM (S/N = 3). Compared with the latest research results on Pt-based catalysts for DA, the developed Pt/CNTs-N-S/GCE exhibits superior performance (Table 1).

The anti-interference ability of electrochemical sensors is of crucial importance. We assess this characteristic using the I-t curve method. The experimental operation involves sequentially injecting interfering substances, such as DA, into a 0.2 M PBS saline, and then recording the corresponding I-t curves. As shown in Figure 5c, the current rose rapidly after the addition of DA. There was no significant change after the addition of the interfering substance. After adding DA again, the current rose rapidly and stabilized, proving that Pt/CNTs-N-S has good sensitivity and a stable response to DA. Even in the presence of interfering substances that are 10 times higher than the DA concentration, such as ascorbic acid (L-AA, 3 mM), uric acid (UA, 3 mM), and glucose (GLU, 3 mM), the current change was negligible, highlighting its outstanding anti-interference performance. Although UA caused a slight increase in the current, the impact was negligible and could be ignored. The response values of the interfering substances are shown in Figure 5d. The results indicate that the sensor we have fabricated has a good anti-interference ability.

### 3.3. Analysis of Sensitivity, Reproducibility and Stability Performance

**Figure 6:**
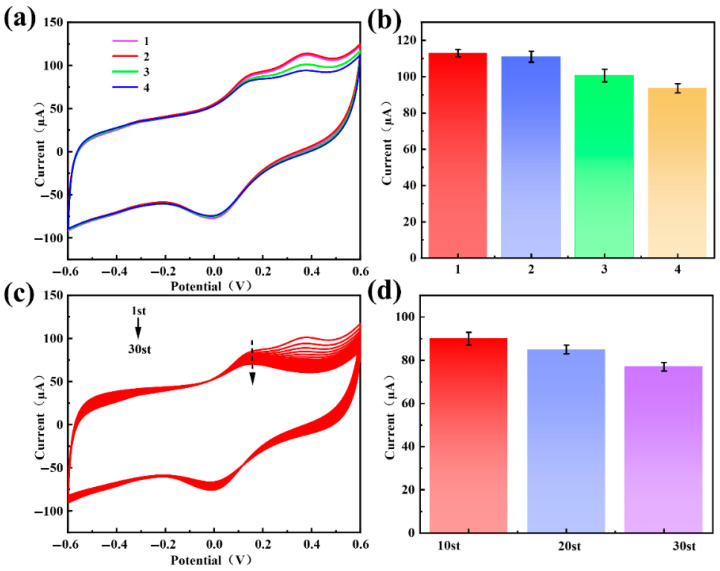
(**a**) CV curves and (**b**) current values obtained from four independent Pt/CNTs-N-S/GCE; (**c**) cyclic voltammetry curves and (**d**) current values obtained from cycle tests.

Repeatability and stability are two crucial characteristics of electrochemical sensors. To evaluate the repeatability of Pt/CNTs-N-S, four parallel experiments were conducted. The experimental results showed no significant variation. Current values consistently remained around 112 μA, with a relative standard deviation of 7.5% (Figure 6a,b), indicating excellent repeatability. Based on the experimental data in Figure 6c,d, after 30 cycles of experiments, the current value decreased by only 9.2%, indicating good experimental stability. These results are derived from three independent parallel experiments, verifying the reliability and repeatability of the data.

### 3.4. Mechanism Analysis of DA Detection by Pt-CNTs-S-N Electrochemistry

The sensitivity of electrochemical sensors is influenced by the electrochemically active surface area (ECSA) and active sites on the electrode surface [42,43]. A larger ECSA means more active sites, which can accelerate the oxidation–reduction reactions of the target analyte and enhance the catalytic efficiency [44,45,46]. The Pt/CNTs-N-S electrochemical sensor, with its composite nanostructure, combines the catalytic activity of Pt with the conductive property of nitrogen–sulfur-*co*-doped carbon nanotubes, optimizing the catalytic ability and mass transfer efficiency of the electrode surface, thereby enabling efficient and sensitive detection of dopamine. This design synergistically combines the advantages of each component: CNTs provide high specific surface area and electron transport pathways; N and S doping introduces catalytic sites and modulates electron distribution; and Pt nanoparticles further enhance the electrode’s catalytic activity and selectivity. DA molecules are adsorbed onto the surface of the Pt/CNTs-N-S-modified electrode. The S- and N-doped CNTs effectively adsorb DA molecules through chemical bonding and electrostatic interactions [47,48]. Furthermore, based on the experimental results, a mechanism was inferred. The presence of Pt nanoparticles enhanced the adsorption capacity of DA. At the same time, Pt formed stable coordination bonds with the phenolic hydroxyl groups of DA [49,50]. Its anti-interference ability is attributed to the synergistic effect between Pt and the sulfur- and nitrogen-doped carbon nanotubes.

**Table 1:** Comparison of different modified electrodes.

Modified Electrode	Method	Linear Range	Detection Limit	Reference
NiMoO_4_@CNTF/GCE	CV	0.1–1.2 mM	58.2 μM	[51]
Cu/Cu_x_O/PGEs	DPV	0.3–53 μM	1.06 μM	[52]
MWCNT-Spng/SPCE	CV	0.2–0.95 mM	0.83 μM	[53]
CuO:Mn/SPE	DPV	1–100 μM	30.3 μM	[54]
Flexible FcCH_2_OH/GOx/MWCNT-SPE	DPV	0–200 μM	31.7 μM	[55]
TM-CNT600/GCE	DPV	0–25 μM	1.42 μM	[56]
Ni-ZIF-8/NS-CNTS/CS	DPV	8–500 μM	0.93 μM	[57]
**Pt/CNTs-N-S/GCE**	**DPV**	**0.0078–2 mM**	**0.73 μM**	**This work**

### 3.5. Detection in Actual Samples

By detecting the content of DA in the serum, the applicability of the Pt/CNTs-N-S sensor to actual samples was evaluated. Different concentrations of DA standard samples were added to the PBS solution containing serum (0.2 M, pH = 7.0). The results are shown in Table S2. The recovery rate was between 99–103.3%. The experiment demonstrated that Pt/CNTs-N-S can accurately detect the content of DA in actual samples. However, our study has certain limitations. Specifically, if we want to describe the physiological range of dopamine in the human body, the detection range (0.0078–2 mM) and detection limit (0.73 μM) of the sensor we prepared would face significant challenges. Additionally, the presence of neurotransmitters in the human body would also interfere with the detection.

## 4. Conclusions

In this study, the constructed Pt/CNTs-N-S exhibited excellent detection performance and anti-interference ability in the electrochemical detection of DA. The linear current response range and the detection limit were 0.0078–2 mM and 0.73 μM (S/N = 3). When interfering substances such as L-ascorbic acid (L-AA), uric acid (UA), and glucose (Glu), at 10 times the concentration of DA, were added, the current value did not show any significant change. The improved performance of the Pt/CNTs-N-S/GCE sensor is attributed to the introduction of S and N dopants, which optimized the structure and surface properties of the CNTs carrier, and the dispersion and electrochemical surface area of these Pt nanoparticles. This sensor enables the quick, easy, and selective detection of DA while also providing a novel platform for biosensor applications.

## Data Availability

The original contributions presented in this study are included in the article.

## Supplementary Materials

The following supporting information can be downloaded at: https://www.mdpi.com/article/10.3390/s26061879/s1, Figure S1: EDX analysis of Pt/CNTs-N-S; Figure S2: The TEM (a–c) and mapping diagrams (d–f) of Pt/CNTs-N; Figure S3: The TEM (a–c) and mapping diagrams (d–f) of Pt/CNTs-S; Figure S4: CV curves of Pt/CNTs-N-S, Pt/CNTs-N, Pt/CNTs-S, Pt/CNTs and Pt/C in 0.5 M H_2_SO_4_; Figure S5: The Epa and Epc curves of Pt/CNTs-N-S at different sweep rates; Figure S6:The Ipa and Ipc curves of Pt/CNTs-N-S at different sweep rates; Table S1: The results of these mateirals measured by XPS; Table S2:Determination of DA in human serum samples (n = 3).
