# Supplementary material for: A Novel Dopamine Electrochemical Sensor Based on Pt/CNTs-N-S/Electrode
Source: Sensors (Basel). 2026 Mar 17;26(6):1879. doi: 10.3390/s26061879 (PMC13030083; doi:10.3390/s26061879)
Supplement: Supplementary file 1 [file sensors-26-01879-s001.zip › sensors-4097614-supplementary.pdf]

Support information

A novel dopamine electrochemical sensor based on Pt/CNTs-N-S/electrode

Pingping Yang[1], Zhaopu Li[1], Jinpu Xie[2], Yukun Tang[2], Yinchen Liu[4], Lingxin Zhou[4] Tengfei Duan[3], Zhonghui Deng[1], Siwen Du[1], Qifei zhang[1], Yabing Lu[1], Jingjing Du[2*], Lijian Xu[1*]

[1] School of Biological Science and Medical Engineering, Hunan University of Technology, Zhuzhou 412007, China

[2] School of Packaging Engineering, Hunan University of Technology, Zhuzhou 412007, China

[3] College of Science and Technology, Hunan University of Technology, Zhuzhou 412007, China

[4] School of Materials Science and Engineering, Hunan University of Technology, Zhuzhou 412007, China

*Corresponding author: 10668@hut.edu.cn (J.D.); xlj235@hut.edu.cn (L.X.)

Supplementary Figures and Table

Fig S1. EDX analysis of Pt/CNTs-N-S

Figure S2. The TEM (a-c) and mapping diagrams (d-f) of Pt/CNTs-N

Figure S3. The TEM (a-c) and mapping diagrams (d-f) of Pt/CNTs-S

Figure S4. CV curves of Pt/CNTs-N-S, Pt/CNTs-N , Pt/CNTs-S ,Pt/CNTs and Pt/C in 0.5 M H_2SO_4.

Figure S5. The Epa and Epc curves of Pt/CNTs-N-S at different sweep rates

Figure S6. The Ipa and Ipc curves of Pt/CNTs-N-S at different sweep rates

Table S1. The results of these mateirals measured by XPS

Table S2. Determination of DA in human serum samples (n = 3)

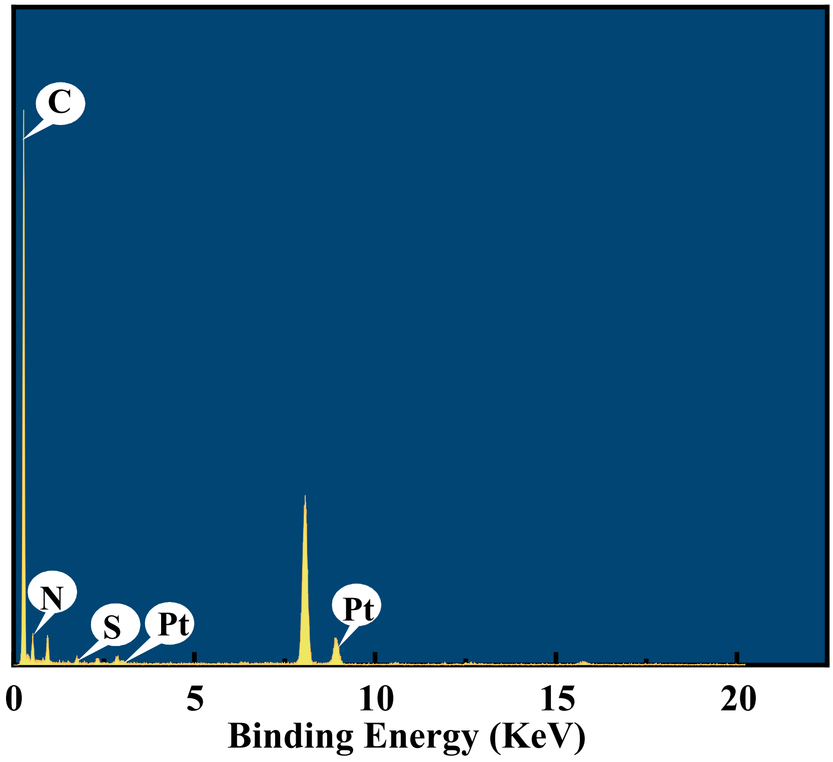

Figure S1. EDX analysis of Pt/CNTs-N-S

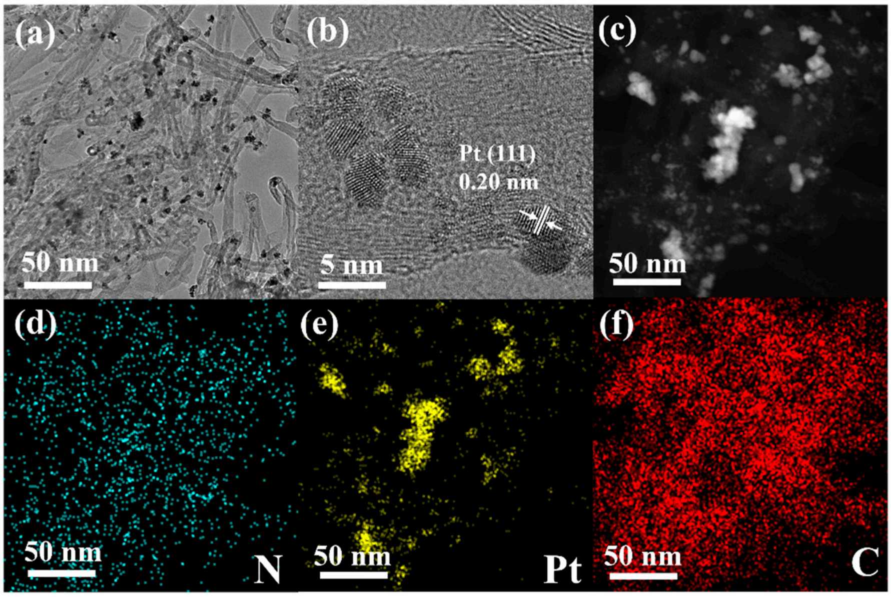

Figure S2.The TEM (a-c) and mapping diagrams (d-f) of Pt/CNTs-N

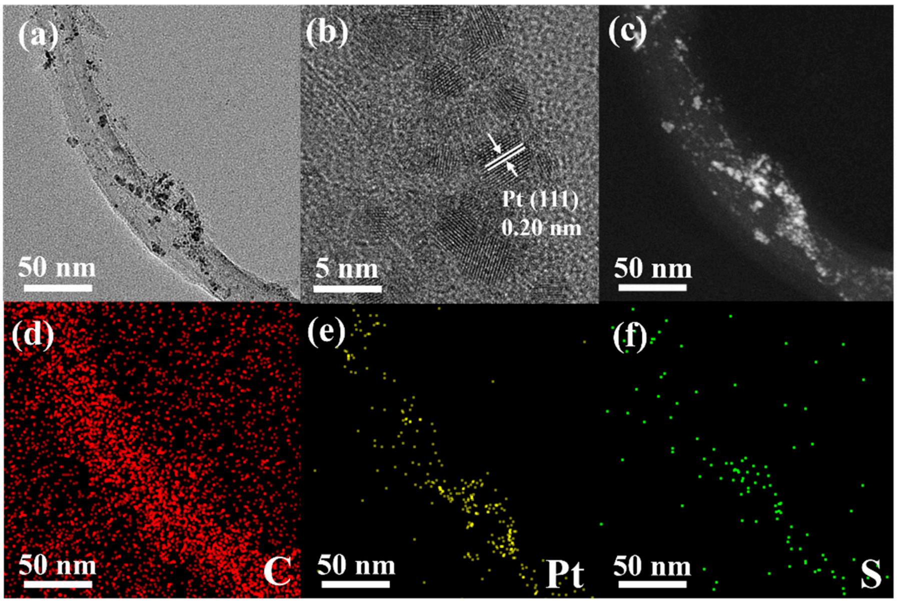

Figure S3.The TEM (a-c) and mapping diagrams (d-f) of Pt/CNTs-S

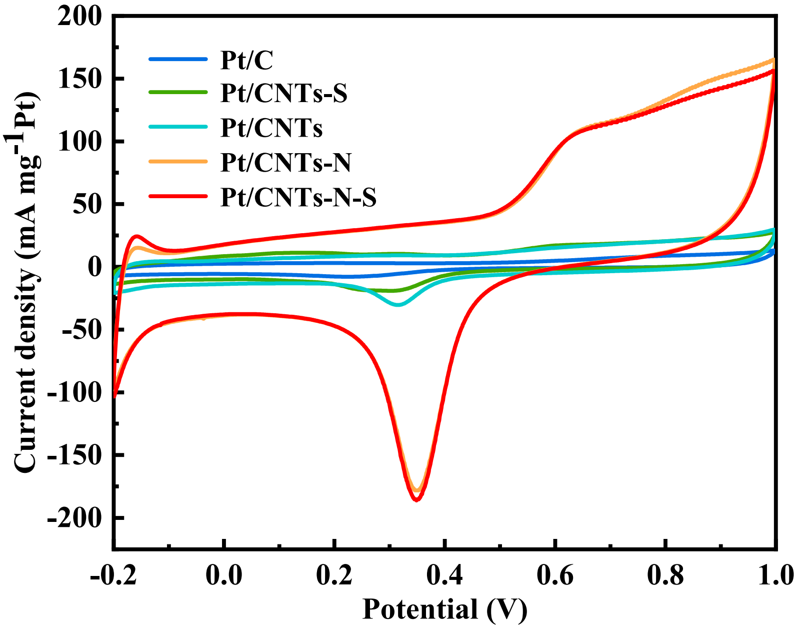

Figure S4. CV curves of Pt/CNTs-N-S, Pt/CNTs-N , Pt/CNTs-S, Pt/CNTs and Pt/C in 0.5 M H_2SO_4.

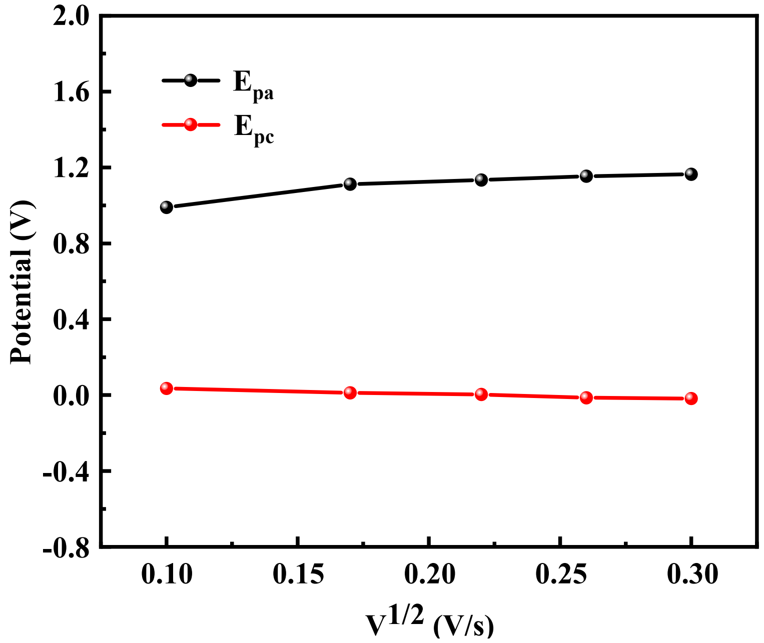

Figure S5. The Epa and Epc curves of Pt/CNTs-N-S at different sweep rates

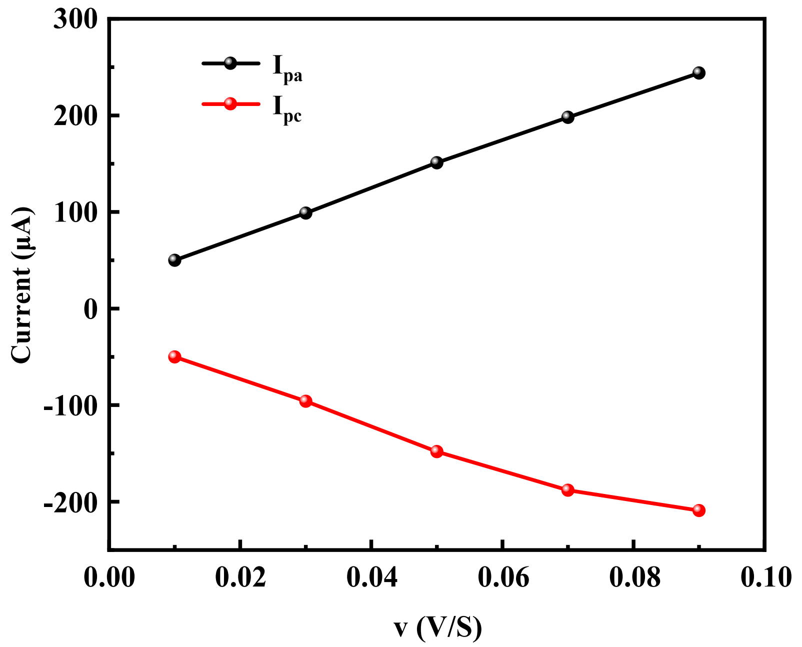

Figure S6. The Ipa and Ipc curves of Pt/CNTs-N-S at different sweep rates

Table S1. The results of these mateirals measured by XPS.

Catalysts	Peaks	Binding energy (eV)	Species	Concentration (%)
Pt/CNTs-N-S	(Pt 4d 5/2)	316.9 eV	Pt(0)	61.19%
	(Pt 4d 3/2)	334.6 eV	Pt(+2)	38.81%
Pt/CNTs-N	(Pt 4d 5/2)	315.3 eV,	Pt(0)	62.22%
	(Pt 4d 3/2)	334.5 eV	Pt(+2)	37.78%
Pt/CNTs-S	(Pt 4d 5/2)	314.8 eV	Pt(0)	62.02%
	(Pt 4d 3/2)	332.4 eV	Pt(+2)	37.98%
Pt/CNTs	(Pt 3d 5/2)	314.7 eV	Pt(0)	62.62%
	(Pt 3d 3/2)	331.4 eV	Pt(+2)	37.28%

Table S2. Determination of DA in human serum samples (n = 3)

	Added (μM)	Founded (nM)	Recovery (%)	RSD(%)
1	0	0	--	--
2	3	3.1	103.3	1.68
3	5	5.02	100.4	0.24
4	7	6.9	99	2.64
